# From a false sense of safety to resilience under uncertainty

**DOI:** 10.3389/fpsyg.2024.1346542

**Published:** 2024-05-27

**Authors:** Matti T. J. Heino, Daniele Proverbio, Kaisa Saurio, Alexander Siegenfeld, Nelli Hankonen

**Affiliations:** ^1^Faculty of Social Sciences, Unit of Social Research, Tampere University, Tampere, Finland; ^2^Faculty of Social Sciences, Discipline of Social Psychology, University of Helsinki, Helsinki, Finland; ^3^Department of Industrial Engineering, University of Trento, Trento, Italy; ^4^New England Complex Systems Institute, Cambridge, MA, United States

**Keywords:** complex systems, attractor landscapes, safety, security, myth of mass panic, behavior change, non-linearity, change processes

## Abstract

Understanding and acting upon risk is notably challenging, and navigating complexity with understandings developed for stable environments may inadvertently build a false sense of safety. Neglecting the potential for non-linear change or “black swan” events – highly impactful but uncommon occurrences – may lead to naive optimisation under assumed stability, exposing systems to extreme risks. For instance, loss aversion is seen as a cognitive bias in stable environments, but it can be an evolutionarily advantageous heuristic when complete destruction is possible. This paper advocates for better accounting of non-linear change in decision-making by leveraging insights from complex systems and psychological sciences, which help to identify blindspots in conventional decision-making and to develop risk mitigation plans that are interpreted contextually. In particular, we propose a framework using attractor landscapes to visualize and interpret complex system dynamics. In this context, attractors are states toward which systems naturally evolve, while tipping points – critical thresholds between attractors – can lead to profound, unexpected changes impacting a system’s resilience and well-being. We present four generic attractor landscape types that provide a novel lens for viewing risks and opportunities, and serve as decision-making contexts. The main practical contribution is clarifying when to emphasize particular strategies – optimisation, risk mitigation, exploration, or stabilization – within this framework. Context-appropriate decision making should enhance system resilience and mitigate extreme risks.

## Introduction

Organizations, decision-makers and researchers usually assume stability or merely gradual changes of their operating environment. This implies a focus on optimizing current practices based on past experiences, thus aiming to build a sense of safety while managing proximal risks. While this view may hold in many cases, it neglects crucial aspects, and an exaggerated emphasis on this view of relative stability can lead to an overly rosy image regarding the continuity of operations. If the context – unbeknownst to the actors – in principle allows abrupt shifts or changes into alternative scenarios where they are plausible, the “default” perspective of assumed stability may lead to a false sense of safety. In the most pressing case, high-impact events could lead to a state of irreversible loss (“ruin”) from which recovery is impossible.

In this paper, we discuss the potentially harmful effects of false sense of safety, defined as feeling safe while neglecting the possibility of ruin. We present cases where non-linear changes and the emergence of potential abrupt shifts make irreversible losses plausible, and we suggest an actionable framework to make sense of complex decision-making environments, to mitigate false senses of safety and develop coping and resilient strategies. To this end, we propose four principled guideline sets, and show how to employ them to guide decisions. Recognizing non-linear risks aids in effective risk mitigation, promoting the resilience of social systems. The framework introduced and discussed in this manuscript provides a conceptual basis to better recognize and categorize current states, and identify hot points of intervention.

The notion of a “space of possibilities” offers a valuable framework for comprehending risk and synthesizing existing frameworks from the literature. All possible behavioral states can be visualized as points in a space. Over time, a system may experience various states; this evolution is visualized as movements within such space. Therein, we can further identify areas that are much more regularly explored by the system, and where the system tends to return even after small perturbations that perturb it. We term such limited areas “attractors.” For example, during a pandemic, a society could “reside” in an attractor of *no respirator use*, and then shift to an attractor of *high prevalence of respirators* (Heino et al., 2022). This shift could happen upon crossing a tipping point, which refers to a critical threshold that, once crossed, can lead to a rapid and significant transition from one attractor to another.

We first provide background information about false sense of safety deriving from neglecting the possibility of ruin, and we survey the necessity of complex systems thinking to circumvent the limitations of widespread linear thinking. Then, we illustrate the notion of attractors in the space of possibilities, forming landscapes which are central to fully appreciate the introduced framework. These concepts will be employed to discuss the framework and its implications to decision-making processes, together with selected examples.

## Neglected ruin problems

People often possess an intuitive capacity to apprehend extreme risks, like the sense of disgust associated with infectious disease, which prompts individuals to avoid situations or actions carrying a high risk of infection (Oaten et al., 2009). One way individuals engage in risk avoidance is through the phenomenon of loss aversion, where they show a pronounced tendency to favor avoiding losses over acquiring equivalent gains. In the literature, loss aversion is often labeled as a cognitive bias (Tversky and Kahneman, 1992) and interpreted as an irrational preference; nonetheless, it may reflect a deeper evolutionary logic when viewed in the larger context, e.g., one of cumulative risks (Taleb, 2018, pp. 226–228).

When one only considers phenomena at the micro-scale, the health risk from a single cigarette, or a single exposure to dangerous pathogens may be negligible. But when taking in a wider view, the cumulative impact of even small repeated events can be extreme: Many people being ill in short succession (as with many people smoking many cigarettes) has economical and health consequences that cannot be understood from observing a singular instance. In real-life scenarios, as opposed to controlled settings, risks accumulate, and their potential for catastrophic outcomes cannot be ignored (Taleb, 2007). In fields like insurance and probability theory, the ultimate case of “ruin” and its probability are actively studied, and they refer to situations from which recovery is impossible, such as bankruptcy.

Understanding loss aversion in this light reframes it as a reasonable and even essential risk management strategy. The asymmetric valuation of risks and gains is rational because the consequences of severe losses can extend far beyond the contingent states, potentially leading to a state of ruin. Avoiding such losses, therefore, takes precedence over making equivalent gains, which add value but do not guarantee survival in the long term. This perspective suggests that loss aversion, rather than being merely a cognitive bias, can be a critical aspect of decision-making strategies aimed at ensuring long-term viability in the face of risks that could lead to irrevocable consequences (Taleb, 2018).

On the other hand, downplaying loss-averse dispositions and neglecting the possibility of ruin states, under the assumption that any additional small risk would result in an additive small output, may lead to a false sense of safety – with consequences on societal decisions. In fact, instances of researchers underestimating risks and attributing cognitive biases to others are not uncommon. In the period leading up to the COVID-19 pandemic, several prominent researchers asserted that public concern was unwarranted, attributing it to probability neglect and other biases (Sieroń, 2020). This reflects long-standing erroneous presumptions that people’s risk perception needs to be downplayed, in order to prevent panic (Clarke, 2002), an idea – also termed the “myth of mass panic” – which lacks empirical support and gives rise to policies that undermine the public’s capacity for resilient behaviors (Drury et al., 2013, 2019). For this reason, science-based public communication guidelines recommend establishing realistic expectations rather than fostering unrealistic ones about the situation or its resolution (Tumpey et al., 2019).

## Risk responses in a complex world

The case of risk cumulation does not encompass all possibilities for the existence and possibility of ruin states, as complex systems may be prone to irreversible failure due to several modes (Olsson et al., 2015). In what follows, we refer as “system” to any entity capable of non-trivial behaviors, be it an individual or a collection of interacting individuals as well as political, economic, social, technological, legal, and/or environmental factors. After setting a scope, the same underlying systems principles apply to any scale (Siegenfeld, 2022). For clarity, we constrain our examples to the scope of a society experiencing the threat of a novel pathogen. This scope encompasses multiple scales from individuals to households, neighborhoods, cities, municipalities, and so forth.

Over time, complex systems may spawn unseen and extreme risks. If left unaddressed in an early phase, these can exhibit non-linear behaviors and lead to catastrophic failures (Taleb et al., 2020). To overcome conventional limitations and develop a broader framework to investigate such phenomena, this article proposes a multidisciplinary lens to visualize and understand system dynamics, as well as the risks therein. It is based on insights from complex systems science, adept at understanding non-linear developments and ‘Black Swan’ events,^1^ while offering a fresh perspective on policy formulation. By weaving together insights from managerial practice (Snowden and Boone, 2007; Joint Research Centre, European Commission et al., 2021), risk management (Taleb et al., 2014; Taleb, 2020), and the framework of attractor landscapes in human behavior change (Heino et al., 2022), this work aims to build on calls to craft policies suitable for an interconnected world (Heino et al., 2023).

**Figure 1:**
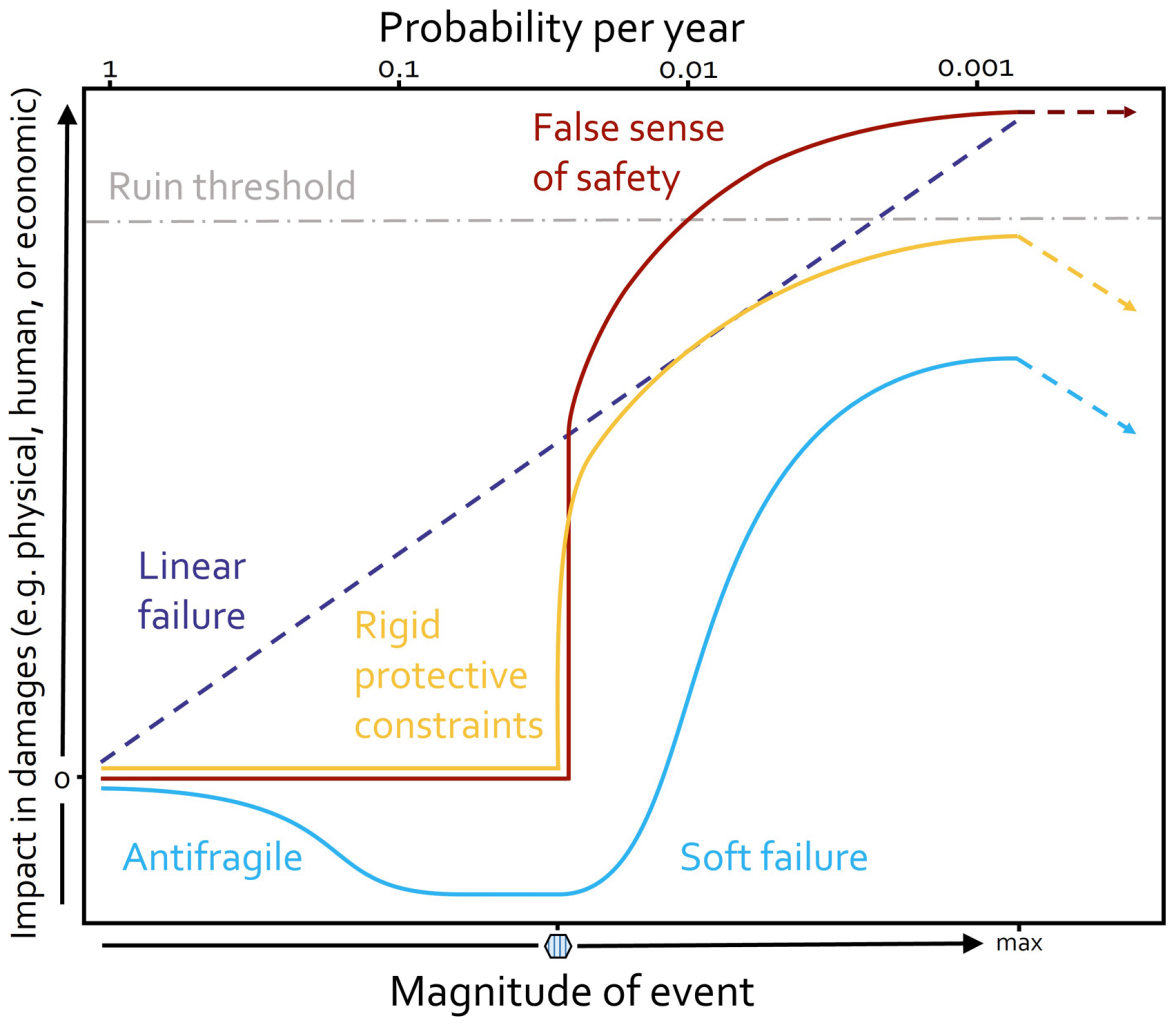
Schematic strategies for damage control, illustrating linear and non-linear impacts of adverse events. Purple: The linear case assumes an outcome that is gradual and directly proportional to the event’s magnitude. Blue: In this case, the system anticipates failure and is therefore capable of coping and mitigating it. Yellow: This line indicates scenarios where a protective barrier holds up to a point, until giving away catastrophically. Red: This line depicts the previous situation, in the worst case, where layers of protection have been omitted. The hexagon on the event magnitude scale marks critical values yielding a “tipping point” (see Figure 2) in the behavior of the non-linear curves, toward a new attractor. Gray: A threshold for a “zone of no return.” Dashed lines with arrows indicate the possibility of recovery by turning toward the original state. This is possible in the blue and yellow case, whereas recovery after passing the ruin threshold – whose exact position may not be known – is not possible. Figure adapted with permission from Bar-Yam and Seguin (2010).

Non-linear responses to events can be of different nature, prompting alternative damage control strategies. A visual representation of how various non-linear responses can emerge in the face of negative incidents is offered in Figure 1. If systems changed according to linear thinking, we would encounter a scenario like in the purple dashed line: any outcome is gradual and proportional to events’ magnitude.

The blue line in the figure illustrates a resilient scenario. The beginning of the line curves downward indicating initial gains from harm, like fixes and overcompensating on uncovered weaknesses. This exposure profile is known as *antifragile* in the literature (Taleb and West, 2023), and can be exemplified by a scenario where a natural disaster causes little damage in a community, but ends up strengthening the members’ social cohesion and disaster preparedness levels. When the antifragile portion of the curve ends, the blue line experiences a “soft” failure (Bar-Yam and Seguin, 2010): this slower shift allows for timely repairs or replacements of protective layers^2^ – before the harm becomes extensive. Examples of such layers from the COVID-19 pandemic include air hygiene (Jimenez et al., 2022), mass testing (Philippe et al., 2023), masks and other effective public health and social measures (Royal Society Expert Working Group, 2023), combined with an inter-and intra-country modularization approach to prevent sudden failure cascades (Scheffer et al., 2012; Oliu-Barton and Pradelski, 2021).

Systems may experience alternative cases. The yellow line depicts a scenario where a protective buffer, akin to a dam’s structural strength or the authority of a law, holds up to a certain threshold magnitude, until it eventually gives way. In this case, no apparent changes occur for low stress (giving rise to a false sense of safety), until a tipping point when an abrupt change occurs, into something potentially irreversible. An example could be the overburdening of a healthcare system that faces a treatable but aggressive novel pathogen; when no more patients can be treated, damages start accumulating rapidly. On top, the red line portrays a situation where, additionally, a population has been assured that there is no reason for concern or preparation; when the damage occurs, lack of communities’ self-organized protective layering leads to more extensive damages. In this case, the system crosses a threshold of ruin, losing all resilience (capacity to absorb stress) and hence the capacity to capitalize on future opportunities (Asmussen and Albrecher, 2010). In these examples, shifts are not gradual, as in the blue line, but take place upon reaching threshold (tipping) points, such as the one depicted by the hexagon in Figure 1. The notion of tipping points is central in our framework and will be inspected in more detail in the next section. In what follows, we will introduce principles to guide organizations and policies through enacting interventions that promote “blue line” scenarios, rather than “yellow or red line” ones.

## Attractors and tipping points in the space of possibilities

As described in the introduction, we can understand system dynamics via attractors and tipping points in a space of possibilities. In this representation, the system can be envisioned as a ball moving within a rugged terrain of valleys and hills. The valleys in the landscape represent attractors – states toward which the system naturally gravitates, like a ball rolling downhill to settle at the bottom of a valley. The deeper the valley, the more stable the attractor state, as it would require a significant perturbation to push the ball out of a deep valley. In contrast, hills in the landscape represent repeller states – unstable regions that the system tends to avoid, like a ball rolling away from the top of a hill.

In the case of no attractors, a push in one direction corresponds to a proportional step, like the purple scenario in Figure 1. If there are equal probabilities of occupying any given tile, over an extended period, each square will be equally visited by the marker. This linear situation – the case of no permanent trapping in attractors or ruin states – is also referred to as “ergodic” in the research literature (Peters, 2019; Heino et al., 2021). On the other hand, movement away from an attractor and across a tipping point exemplifies non-linear changes, because such a transition can lead to abrupt and substantial impacts on the system’s state and well-being, with either positive or negative consequences.

A system can be pushed out of its current attractor state and across a tipping point in two main ways:

1. If the valley becomes shallower over time, losing its “pull,” even a small perturbation can be enough to push the ball over the hill.
2. If the system is hit by an extremely large perturbation – like a black swan event – the ball can be pushed out of even a deep valley and into a new state.

**Figure 2:**
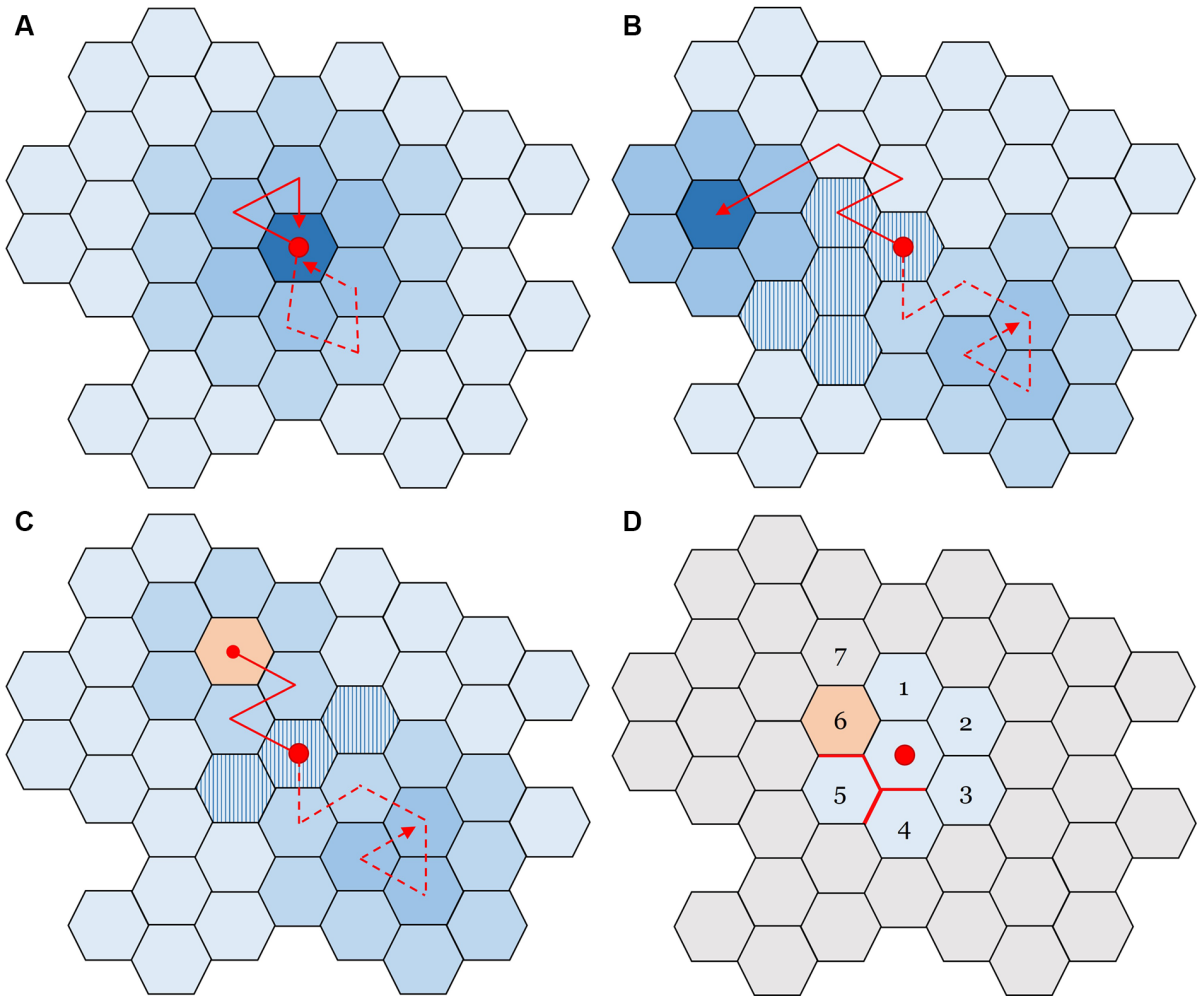
Representations of a space of possibilities, where each tile is a system state. Solid and dashed lines indicate alternative paths that can be explored, stemming from an initial condition marked by the red dot in the center of the space. **(A)** The simple case with only one attractor. After the system is perturbed, it tends to return to the original state. **(B)** A situation with two attractors, whose depth is indicated by darkness of color. They “trap” and hold the system if it enters the basin of attraction (darker blue), making the system less likely – in proportion to attractor depth – to leave after being captured. A tipping point from one attractor to the next occurs when the system residing in one of the attractors crosses the striped tiles. **(C)** Space with one attractor leading to a systemic ruin risk (orange tile); trajectories that land there are permanently halted. The dashed trajectory depicts a route to the “safe” attractor, but the ruin state nonetheless remains as a possibility. **(D)** A space of possibilities, as seen from the policy maker’s perspective: only the “adjacent possible” states can be observed. Gray tiles represent states that are unknown (see text for details). Figure exapted from Lewin (1951, p. 92).

This point of view augments the representation of the scenarios from Figure 1 by adding the notion of “possibilities.” A visual example is shown in Figure 2: each individual tile stands for a potential state the system could be in, while arrows depict evolution (movements). In Figure 2A, only a single attractor exists. Starting from the red dot, the system can be perturbed away from the initial state, but it is expected to quickly return to it.

In Figure 2B, we observe two attractors (dark shades). When the system reaches the edges of one or another area, it is influenced or ‘pulled’ by the attractor. The striped tiles, at the frontier of two attractors, represent a collection of tipping points. Upon reaching it from one of the attractors, the system can shift states rapidly as it flows into the attractor on the other side of the tipping point. An example could be a shift from an unfavorable public opinion toward pandemic control practices, turning quickly into a favorable one. Such change may be prompted by a small minority overturning current belief and hence ‘tipping’ the majority’s stance (Centola et al., 2018).

Figure 2C introduces a ruin state, marked by an orange tile, which the system cannot depart from after accessing it. For example, consider a society, which currently resides in the bottom-right attractor (illustrated by the dashed trajectory). This attractor can be interpreted as the “business as usual” state, while the orange tile would correspond to massive death counts due to a novel pathogen (or, equally, climate change or a war). Instead of gradual decline, a tipping event or drastically reduced resilience may trigger a catastrophic and unexpected shift (like the yellow or red lines in Figure 1).

In public discourse, efforts to mitigate the risk posed by the orange tile might be dismissed as superfluous fearmongering, unnecessarily disturbing the public’s sense of safety, as the risk has never actualized before. A landscape with only one possible attractor feels safe. But it is crucial to understand that, had a transition to ruin happened, the current discourse would not take place – a prime example of survivorship bias in the absence of the aforementioned black swan events (Taleb, 2007; Taleb et al., 2014). The absence of a previous leap from one attractor to a disastrous one by no means negates its potential occurrence in the future. In fact, if this perilous state persists and the attractor does not capture the system permanently, a possible future will eventually encounter it if the original attractor is destabilized, resulting in ruin (Taleb et al., 2014). This highlights the limitations of relying solely on historical data when evaluating phenomena with potential for extreme impact.

Figure 2D illustrates the uncertainty present in real policy-making. Here, the discernible space of possibilities is confined to the “adjacent possible” states, represented by tiles 1–6. The concept of an *adjacent possible*, introduced by Stuart Kauffman and reviewed in Björneborn (2020), alludes to the immediate range of potential future states a system can access from its current state. It represents the evolutionary frontier, highlighting what is immediately feasible given the current circumstances. Of these, tiles 1–5 signify states that maintain the system’s continuity. However, due to the barriers indicated by the red borders, the system presently faces substantial time or energy constraints in accessing tiles 4 and 5. Tile 4 can be accessed via tile 3, representing an “oblique” (Kay, 2012) strategy where desired states are reached indirectly. The gray tiles, labelled as 7, symbolize states that remain undefined – and at least in part unknowable – until the system progresses nearer to them. Those *possibles* may prove to be tipping points for future scenarios.

The space of possibilities is often not static but can be in constant flux; attractors can change position and desirable states become undesirable. In such dynamic and non-stationary environments, strategies anchored in meticulous, extended-term foresight can fail consistently, especially if they embrace time horizons that are much longer than the period of change of the state landscape. This is because the circumstances upon which plans were conceived might be rendered obsolete by the time of their execution. A notable historical instance of this dynamic is evident in the intricate 5-year planning frameworks of the Soviet Union.

Building upon this framework, in the next section we finally present four candidate decision-making principles to navigate through key scenarios characterized by varying uncertainty. We further explore the future implications and applications of the principles derived from the discussion above, and show how to employ them in daily practice.

## Decision-making principles based on attractor landscape types

In this section, we propose a set of plausible attractor landscape forms and we discuss how these can be considered as generic decision making contexts. We do so by associating them with facets of the well-established Cynefin decision making framework, recently featured as the basis of the *European Union Field Guide for Managing Complexity (and Chaos) in Times of Crisis*, henceforth referred to as the EU Field Guide (Snowden and Boone, 2007; Joint Research Centre, European Commission et al., 2021). The baseline landscape form – a landscape of pure stability – presents the most common thinking in organizations. We argue that blind trust in this scenario, without foreseeing its changes into one of the others, is a major source of false sense of safety, which may lead to abrupt damages as seen in Figure 1. Consequently, we propose alternative guidelines, based on the attractor landscape framework, to extend this view, so that systems such as organizations might expand their focus to better include scenarios where merely optimizing past practices is not a reasonable strategy.

### Landscape type 1: one deep and stable attractor

#### Contextual principle: optimize existing opportunities and monitor for changes in the landscape

In this first scenario, the situation at hand is stabilized in an attractor which consists of a limited number of possible states, so there is a decent degree of predictability and repeatability. This corresponds to a picture like in Figure 2A. Given the repeatability, past data can be used to optimize exploitation of current opportunities. Changes in the landscape should be carefully monitored, but too much focus on exploration (on the expense of exploitation) would cause ineffective use of the current resources. Decision principles pertaining to the *ordered* domain of the Cynefin framework can be particularly useful, prescribing categorization or analysis of the situation followed by existing tried-and-true practices and conventional goal-setting.

Managing an annual influenza epidemic is a good example: worse seasons can be handled with approximately the same tools as milder ones. However, an over-reliance on data from recent epidemics might falter when facing a novel or significantly mutated pathogen. During the early stages of the COVID-19 outbreak, many countries expected a pandemic similar to a harsh seasonal influenza, creating a false sense of safety. Hence, optimizing current practice must be accompanied with monitoring to rigorously answer the question “are we still operating within the same attractor, or have important contextual factors changed?”

### Landscape type 2: a fluctuating terrain with several attractors

#### Contextual principle: explore and exapt

If the system can easily switch attractors and the terrain changes in time (like in a dynamic version of Figure 2B), the doing of today may not work tomorrow. Yesterday’s goals can be today’s stray paths, as the context can change abruptly upon reaching tipping points. To avoid this pitfall, it is necessary to do constant exploration, where the decision maker keeps what works and discards what does not. Focusing too much on optimizing existing opportunities (e.g., mandating practices which are efficient for the current environment but difficult to divert from) is likely detrimental, as what worked in one attractor may not work in another. Given the uncertainty, a leader needs to focus on decentralized, team-based decision making processes that produce solutions to *varied* issues, rather than make decisions that directly *solve* particular problems (Bar-Yam, 2006, 2017). If there are repeller states that are important to access, the preferred action is to take steps to “lower the hill.” For instance, the state of *a pathogen being eliminated from an area* can turn from a hill to a valley, if the mean size of regional outbreaks and/or transmission between regions is adequately hindered (Siegenfeld and Bar-Yam, 2020).

The EU Field Guide advises us to *adapt in complexity*, and *exapt*: keeping options open while probing the adjacent possible for their evolutionary potential (Joint Research Centre, European Commission et al., 2021). This means parallel, soft-failure probing, in a trial-and-error manner that possibly recombines existing resources, and constantly monitors for unexpected side effects. In doing so, positive spill-overs should be readily amplified, and negative ones dampened (Snowden, 2011, p. 224).

### Landscape type 3: presence of a “ruin” state

#### Contextual principle: cut the downside

A system facing the possibility of total destruction (exemplified in Figure 2C) must prioritize mitigating this risk. This task can be challenging due to the human inclination to attend to tangible and salient issues at the expense of more abstract ones, encapsulated by Kahneman’s (2011, p. 85) *“What You See Is All There Is (WYSIATI)”* principle. Political advocacy for preventative measures against unprecedented disasters can hence be difficult and proactive actions – preventing harm that has not happened – are less tangible than reactive solutions to remove harm that is already visible to the public.

Particularly in this scenario, many observers need to interpret risk-related information from different perspectives. While it is important in preceding scenarios, too, here it is essential to actively involve communities in the decision-making process. By adopting approaches that encourage collective understanding and exploration of potential scenarios, we can encourage numerous parallel grassroots initiatives that shift the landscape of, e.g., norms and attitudes (Snowden, 2024), making ruin less accessible. The intention is to obliquely facilitate implementing policies to address catastrophic risks. To counteract the WYSIATI bias, decision makers could also utilize the power of narrative, engaging the public to craft compelling stories that vividly depict potential future crises. In some endeavors, e.g., insurance may be a feasible option.

### Landscape type 4: high uncertainty on the precipice of ruin

#### Contextual principle: stabilize and constrain

In situations like Figure 2D, with large uncertainties on the landscape morphology, all kinds of unforeseen events might take place when constraints on the system’s behavior are not in effect. The decision maker addresses extreme uncertainty by focusing on stabilization, creating attractors to prevent erosion or disintegration of the system.

The management principle depicted in the EU Field Guide is “addressing chaos”: stabilizing the situation via drastic constraints, to create time for assessment while maximizing optionality (Joint Research Centre, European Commission et al., 2021). As discussed earlier, reacting by lowering *risk perception* with the aim of mitigating an imaginary mass panic, may not improve resilience but rather create a vulnerability for further destabilization. An example of good practice would be New Zealand closing its borders during the early phases of the COVID-19 pandemic. It retained the option to wait for vaccine data from other countries, while managing to protect its citizens from both economic and human losses (Baker et al., 2020; Msemburi et al., 2022; Philippe et al., 2023).

## Future directions

To address the usefulness of the aforementioned principles in their associated contexts, several steps should be taken. First of all, researchers need to link the attractor landscapes built via sense-making exercises to those enacted by mathematical rigor and scientific modeling. Relatedly, numerical assessments of stability could be tested; some proposals for methodology are already available (Heino et al., 2022) and can be built upon.

Complementing experiential sense-making with formal models allows the rigorous testing and comparison of scenarios (Hepburn and Andersen, 2021). One approach to mathematically representing the attractor landscape could involve employing dynamic system models, capturing the shifts and evolutions in real-time. However, challenges might arise in parameterizing these models given the vast array of potential variables. Novel interdisciplinary efforts between modelers and social science scholars could create fruitful synergies.

Usability of the proposed framework should be assessed in collaboration with decision makers, after they have been trained to understand the basic notions. For instance, attractor landscapes were introduced in a novel training program recently piloted for public servants, with the aim to produce the necessary capacity of policy makers to understand and act in complex social systems (Hankonen et al., 2023). Initial feasibility testing among Finnish public servants demonstrated the program’s potential for broad applicability across different policy sectors. Future research should explore ways to encourage, co-research, co-develop, and facilitate the application of these theory-based principles in real-world contexts.

## Discussion

Recognizing non-linear risks aids in effective risk mitigation, promoting the resilience of social systems. The flow-in-landscape framework introduced and discussed in this manuscript provides a conceptual basis to better navigate and categorize current and likely future states, as well as to identify key points of fragility and intervention. For instance, it clarifies that in an evolving global landscape, even evidence-based policy frameworks relying on historical data might struggle to anticipate and address unforeseen challenges and should therefore be augmented. To this end, the manuscript provides a macroscopic description of decision-making principles, according to realistic classes of plausible scenarios, augmenting previous linear viewpoints to incorporate complex nuances.

In public policy, leveraging these principles can enhance both safety and adaptability. For local officials, this may mean deeper community engagement; at broader scales, it suggests a revision of protocols for greater agility against complex issues. One significant challenge is cognitive inertia among decision-makers, which can be potentially addressed through diversifying viewpoints and interactions, ongoing education, as well as promoting adaptability (Heino et al., 2023).

As we navigate risks and opportunities amidst an era of unparalleled complexity, insights from complex systems and psychological sciences can cover blindspots in conventional decision making. This has the potential to move us from a false sense of safety to develop risk identification and mitigation plans for sustainable and persistent safety, increasing resilience and prompt interventions in the face of uncertainty.

## Data availability statement

The original contributions presented in the study are included in the article/supplementary material, further inquiries can be directed to the corresponding author.

## Author contributions

MH: Conceptualization, Methodology, Project administration, Visualization, Writing – original draft, Writing – review & editing. DP: Supervision, Visualization, Writing – review & editing. KS: Writing – review & editing. AS: Writing – review & editing. NH: Funding acquisition, Resources, Supervision, Writing – review & editing.

## Footnotes

^1^‘Black Swan’ events, as introduced by Taleb (2007), are unpredicted yet impactful occurrences that can overturn conventional wisdom.

^2^See also the “Swiss cheese model” of risk management (Shabani et al., 2023).
